# An Uncommon Presentation Severe Hypercalcemia Linked to Cystic Parathyroid Adenoma Despite Negative Sestamibi Scan: A Case Report

**DOI:** 10.31729/jnma.9170

**Published:** 2025-07-31

**Authors:** Smriti Acharya, Sharon Daniel, Suyash Acharya, Rajinder Gupta, Daniel Kannappan

**Affiliations:** 1 Department of Diabetes and Endocrinology, Wrightington, Wigan and Leigh NHS Foundation Trust, Wigan Lane, Wigan, United Kingdom

**Keywords:** *adenoma*, *hypercalcemia*, *hyperparathyroidism*, *parathyroid*

## Abstract

Cystic parathyroid adenomas are uncommon occurrences, constituting approximately 1-2%, of primary hyperparathyroidism cases. They frequently pose diagnostic difficulties due to their limited detection in sestamibi scans. These cases of primary hyperparathyroidism commonly manifest with hypercalcemic crisis despite negative scan results. We present a rare scenario involving an elderly male who arrived at the emergency department with a swollen left neck, primarily due to a thyroid cyst causing breathing difficulties. Incidentally, hypercalcemia was also discovered during examination. Thorough investigations ensued, leading to the decision to perform a right hemithyroidectomy to address the large thyroid cyst and a parathyroidectomy. Histopathological analysis confirmed the presence of a cystic parathyroid adenoma, with subsequent normalization of calcium levels. Following the procedures, the patient experienced hypocalcemia and required calcium supplementation. This case stands out due to the high levels of parathyroid hormone, which typically raises suspicion for parathyroid carcinoma, making it a unique diagnostic challenge.

## INTRODUCTION

Primary hyperparathyroidism arises from heightened activity of the parathyroid glands, leading to elevated calcium levels and diminished phosphate levels. In most instances, the condition is attributed to a solitary, solid adenoma. However, a distinctive subset involves cystic parathyroid adenomas, which emerge through the degeneration of pre-existing adenomas.^1,2^

Cystic parathyroid adenoma is a rare anomaly affecting the parathyroid glands, characterized by either functional or nonfunctional attributes.^1,2^ It’s important to recognize that this condition accounts for only a small fraction, approximately 10%, of all neck masses.^1,2^ In primary hyperparathyroidism, cystic adenomas may trigger severe hypercalcemia despite negative imaging, often resembling parathyroid cancer. This makes diagnosis challenging and requires a high index of suspicion for proper management.^3^

This scenario revolves around a 71-year-old man, previously in good health, who incidentally discovered he had high calcium levels during tests for a thyroid nodule on his right side. He experienced a hypercalcemic crisis and has undergone surgery to remove part of his thyroid and a parathyroid gland. As a result, his calcium levels have returned to normal.

## CASE REPORT

An elderly man was admitted after routine tests showed high calcium and markedly elevated PTH. He had a longstanding right thyroid cyst with tracheal deviation but no symptoms. Routine blood tests revealed high calcium levels and very elevated levels of parathyroid hormone, which seemed inappropriate for a typical parathyroid adenoma. Consequently, he was admitted to the hospital and treated with intravenous Zoledronic acid and fluids to lower his calcium levels. However, despite the treatment, his calcium levels remained persistently high, leading to the initiation of Tablet Cinacalcet therapy, gradually increasing the dosage to the maximum. Unfortunately, despite these interventions, the patient’s calcium levels could not be normalized.

In this case, severely elevated serum calcium 3.28 mmol/l and parathyroid hormone (PTH) 90.9 pg/ml raised suspicion for parathyroid carcinoma over typical hyperparathyroidism. However, nuclear and CT scans were unremarkable, and Fine Needle Aspiration Cytology (FNAC) was inconclusive, making diagnosis challenging.

Due to uncontrolled calcium levels, the patient was referred to otorhinolaringology (ENT). Emergency surgery revealed the cyst originated near the parathyroid, trachea, and esophagus— not the thyroid. A right hemithyroidectomy, parathyroidectomy, and cyst excision were performed, highlighting the case’s complexity.

Firstly, parathyroid carcinoma was suspected due to the patient’s elevated levels of parathyroid hormone and calcium. However, the alternative diagnosis was cystic parathyroid adenoma, which is a benign growth of the parathyroid gland that can also lead to elevated hormone and calcium levels.

CT revealed a large right thyroid cyst (54×88 mm) with retrosternal extension and tracheal deviation, but no signs of malignancy or parathyroid abnormalities. Biopsy showed hemorrhagic fluid (U2), and a parathyroid scan was negative for adenoma. These diagnostic procedures provided crucial insights into the nature of the patient’s condition, aiding in the ongoing assessment and management process.

**Figure 1:**
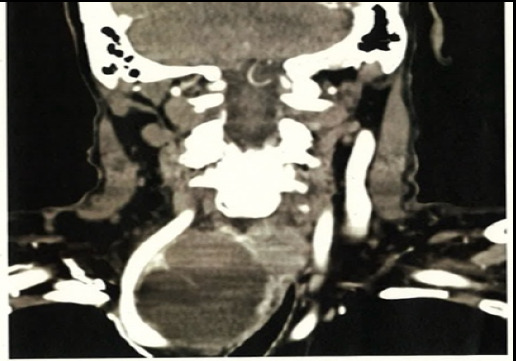
A large right thyroid cyst (54×88 mm) with retrosternal extension.

Following the surgery, the patient developed hypocalcemia (from 3.07 mmol/L to 1.97 mmol/L), with PTH levels returning to normal (from 90.9 pg/ml to 5.3 to pg/ml). Histology confirmed a cystic parathyroid adenoma, positive for AE1/A3 and chromogranin A, and negative for malignancy markers. Lymph nodes were clear, and the thyroid showed only benign nodular changes.

Surgery effectively relieved the patient’s symptoms and normalized calcium and PTH levels. Postoperatively, he developed hoarseness due to likely recurrent laryngeal nerve injury and transient hypocalcemia, managed with supplements. Voice has slightly improved, with no aspiration and stable recovery.

The patient was discharged in stable condition with good biochemical recovery. He will have regular endocrine follow-ups to monitor hormone levels and ENT review for voice changes. He has resumed normal activities and will undergo routine imaging in the coming months.

## DISCUSSION

Primary hyperparathyroidism affects about 21 per 100,000 people annually, with 85% caused by a single adenoma. Rarely, 1-2% involve cystic parathyroid degeneration, which can also lead to hyperparathyroidism. Thus, while solitary parathyroid adenomas are the primary culprit in most instances of primary hyperparathyroidism, the recognition of cystic degeneration as a rare but significant contributor underscores the diverse manifestations of this disorder.^4,5^

Cystic parathyroid lesions are hard to detect, often missed on sestamibi scans. Most cases occur between ages 30-60, with nonfunctional types more common in females and functional ones slightly more in males, adding to diagnostic complexity.^6,7^

According to a study led by Nicholas J. Rivers et al., parathyroid cysts can range from mild hyper parathyroid symptoms like fatigue to compressive signs such as neck swelling, dysphagia, or breathing difficulty, reflecting their varied clinical presentation.^8-11^

Parathyroid cysts are often found incidentally during evaluations for other conditions like primary hyperparathyroidism. Though patients may be asymptomatic, these lesions can lead to serious complications, including hypercalcemic crises or, rarely, malignancy.^12^ It is important to differentiate parathyroid adenoma from other cystic lesions since they can lead to severe hypercalcemic crises and can even develop into carcinoma.

This study has several limitations that merit further exploration and discussion. Firstly, it is important to acknowledge that this was a single case and thus, the findings may benefit from validation through larger case studies for comparison.

## CONCLUSION

Cystic parathyroid adenomas often present with high calcium and PTH levels, large size, and a risk of hypercalcemic crisis, while preoperative imaging may be unreliable. FNAC shows promise for localization but needs further validation in better diagnosis and management.
